# Supplementary material for: Exploratory Analysis of Outpatient Visits for US Adults Diagnosed with Lupus Erythematosus: Findings from the National Ambulatory Medical Care Survey 2006–2016
Source: Healthcare (Basel). 2022 Aug 31;10(9):1664. doi: 10.3390/healthcare10091664 (PMC9498556; doi:10.3390/healthcare10091664)
Supplement: Supplementary file 1 [file healthcare-10-01664-s001.zip › healthcare-1856741-supplementary.pdf]

Table S1. List of all diagnosis codes used for comorbidities classifications (ICD-9 and ICD-10 CM codes).

Disease module	Disease codes (ICD-9)	Disease codes (ICD-10)
Infectious And Parasitic Diseases	001-139	A00-B99
Neoplasms	140-239	C00-D49
Endocrine, Nutritional and Metabolic Diseases, And Immunity Disorders	240-279	E00-E89
Diseases of the Blood and Blood-Forming Organs and Certain Disorders Involving the Immune Mechanism	280-289	D50-D89
Mental, Behavioral and Neurodevelopmental Disorders	290-319	F01-F99
Diseases of the Nervous System and Sense Organs	320-389	G00-H95
Diseases Of the Circulatory System	390-459	I00-I99
Diseases Of the Respiratory System	460-519	J00-J99
Diseases Of the Digestive System	520-579	K00-K95
Diseases Of the Genitourinary System	580-629	N00-N99
Complications Of Pregnancy, Childbirth, and the Puerperium	630-679	O00-O9A
Diseases of the Skin and Subcutaneous Tissue	680-709	L00-L99
Diseases Of the Musculoskeletal System and Connective Tissue	710-739	M00-M99
Congenital Anomalies	740-759	Q00-Q99
Certain Conditions Originating in the Perinatal Period	760-779	P00-P96
Symptoms, Signs and Abnormal Clinical and Laboratory Findings, Not Elsewhere Classified	780-799	R00-R99
Injury, Poisoning and Certain Other Consequences of External Causes	800-999	S00-T88
Supplementary Classification of Factors Influencing Health Status and Contact with Health Services	V01-V91	Z00-Z99

Using ICD-9 and ICD-10 codes and grouped by Multi-level Clinical Classification Software (CCS) categories. Source: Healthcare Cost and Utilization Project (HCUP).

Table S2. Top 10 most frequently mentioned medications for visits of lupus patients over time.

	2006–2010 (n = 10,378,266)			2011–2016 (n = 16,804,929)			P-value[c]
**Rank**	**Medication[b]**	**Frequency[a]**		**Medication[b]**	**Frequency[a]**		
**1**	Prednisone	2,005,926	19%	Hydroxychloroquine	6,216,056	37%	*<0.05**
**2**	Hydroxychloroquine	1,910,098	18%	Prednisone	4,153,175	25%	*<0.05**
**3**	Multivitamin	864,744	8%	Multivitamin	2,892,781	17%	0.09
**4**	Esomeprazole	692,747	7%	Furosemide	2,211,628	13%	0.06
**5**	Methotrexate	691,950	7%	Folic Acid	1,468,899	9%	0.22
**6**	Levothyroxine	449,961	4%	Levothyroxine	1,359,390	8%	0.14
**7**	Amlodipine	447,968	4%	Aspirin	1,275,100	8%	0.14
**8**	Folic Acid	405,497	4%	Methotrexate	1,240,753	7%	0.13
**9**	Alprazolam	365,153	4%	Mycophenolate mofetil	1,085,546	6%	*<0.05**
**10**	Cyclosporine	347,251	4%	Omeprazole	979,839	6%	0.12

aDenominator for proportions is total visits of lupus patients during that time period.
bMedications included generic and brand formulations.
cP-values obtained from Rao–Scott chi-square test of independence with second order adjustment; p-value compares 2006–2010 vs. *2011–2016*
**Significance <0.05.*

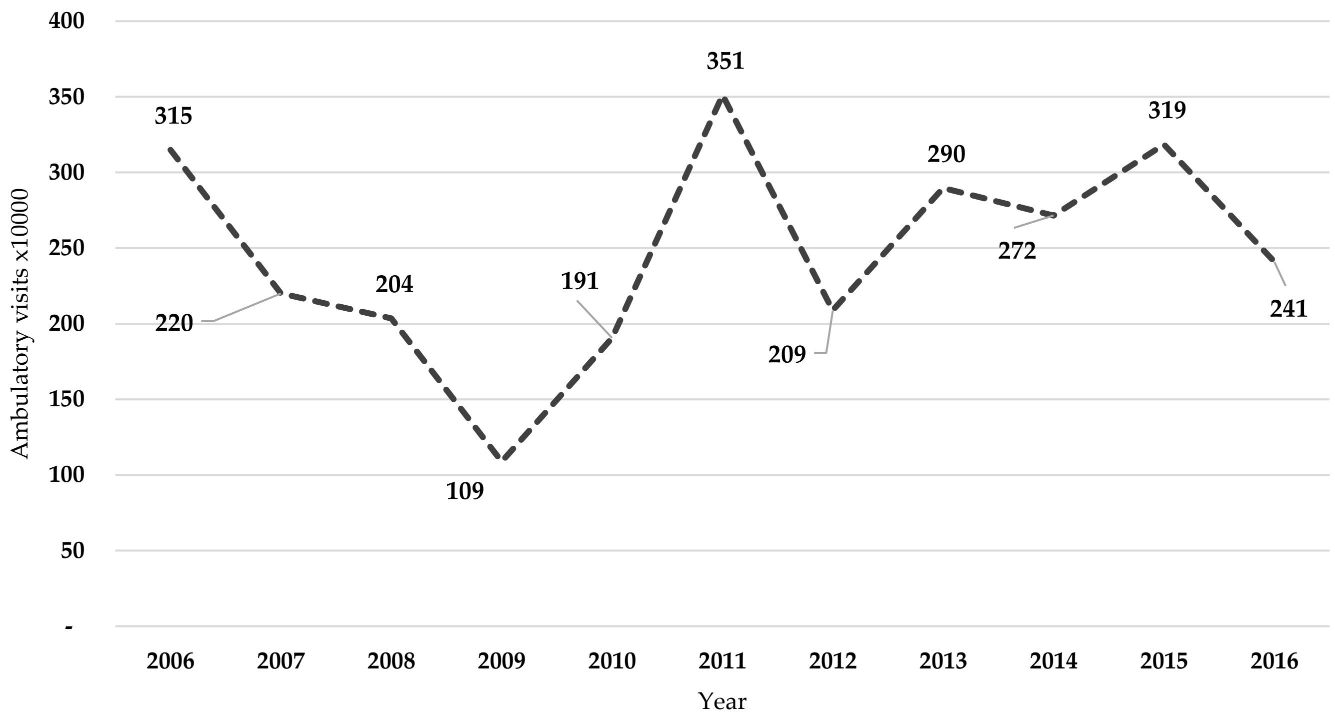

Figure S1. National trends in ambulatory visits for lupus patients in the U.S. from 2006 to 2016. Ambulatory visits are national weighted estimates produced using survey weights, where each patient encounter is assigned an inflation factor called patient visit weight used to predict the total number of physician office visits performed in the U.S.
